# Lipidomic Profiling of Ipsilateral Brain and Plasma after Celastrol Post-Treatment in Transient Middle Cerebral Artery Occlusion Mice Model

**DOI:** 10.3390/molecules26144124

**Published:** 2021-07-07

**Authors:** Maozhu Liu, Mengyuan Chen, Ying Luo, Hong Wang, Haifeng Huang, Zhe Peng, Miaomiao Li, Huizhi Fei, Wen Luo, Junqing Yang

**Affiliations:** Chongqing Key Laboratory of Biochemistry and Molecular Pharmacology, College of Pharmacy, Chongqing Medical University, Chongqing 400016, China; maozhu2021@126.com (M.L. Maozhu~Liu); cmy116165@126.com (M.C.); 100894@cqmu.edu.cn (Y.L.); 101832@cqmu.edu.cn (H.W.); huanghf31@163.com (H.H.); pengzsunshine@163.com (Z.P.); 15320497853@163.com (M.L. Miaomiao~Li); feihuizhi198863@163.com (H.F.); qq.xiao.wen@163.com (W.L.)

**Keywords:** cerebral ischemia/reperfusion injury, celastrol, lipidomics, neuroprotection, UPLC/MS

## Abstract

Celastrol, a pentacyclic triterpene isolated from the traditional Chinese medicine Tripterygium wilfordii Hook. F., exhibits effectiveness in protection against multiple central nervous system (CNS) diseases such as cerebral ischemia, but its influence on lipidomics still remains unclear. Therefore, in the present study, the efficacy and potential mechanism of celastrol against cerebral ischemia/reperfusion (I/R) injury were investigated based on lipidomics. Middle cerebral artery occlusion (MCAO) followed by reperfusion was operated in mice to set up a cerebral I/R model. TTC staining and TUNEL staining were used to evaluate the therapeutic effect of celastrol. Ultra-performance liquid chromatography-tandem mass spectrometry (UPLC/MS) was employed for lipidomics analysis in ipsilateral hemisphere and plasma. Celastrol remarkably reduced cerebral infarct volume and apoptosis positive cells in tMCAO mice. Furthermore, lipidomics analysis showed that 14 common differentially expressed lipids (DELs) were identified in brain and five common DELs were identified in plasma between the Sham, tMCAO and Celastrol-treated tMCAO groups. Through enrichment analysis, sphingolipid metabolism and glycerophospholipid metabolism were demonstrated to be significantly enriched in all the comparison groups. Among the DELs, celastrol could reverse cerebral I/R injury-induced alteration of phosphatidylcholine, phosphatidylethanolamine and sulfatide, which may be responsible for the neuroprotective effect of celastrol. Our findings suggested the neuroprotection of celastrol on cerebral I/R injury may be partially associated with its regulation of lipid metabolism.

## 1. Introduction

Stroke is a serious disease with high mortality and disability rates worldwide. In China, stroke is the leading cause of death and its prevalence and incidence are increasing [1]. The risk of stroke increases with age, but can occur at any age. Risk factors, such as tobacco and work-related productivity, can contribute to stroke in young people [2,3]. Therefore, it is worthwhile to study stroke in young age. Of all strokes, approximately 70 to 80% of cases are ischemic stroke characterized by cerebrovascular occlusion and blood supply insufficiency, and the remaining strokes caused by cerebrovascular rupture are hemorrhagic stroke [4]. For ischemic stroke, rapid restoration of blood flow to the brain with intravenous thrombolysis or endovascular thrombectomy can help reduce the ischemic brain injury. However, the paradox is that the blood recovery may exacerbate brain damage due to various reasons, which is called cerebral ischemia/reperfusion (I/R) injury [5]. At present, drugs used to reduce cerebral I/R injury are very limited in clinical settings. Hence, there is an urgent need to develop highly effective and low toxic therapeutic drugs for the treatment of cerebral I/R injury.

Traditional Chinese medicines (TCMs) have been used for the treatment of cerebrovascular diseases such as stroke for more than two thousand years [6]. Currently, based on the neuroprotective effects of TCMs, the development of new effective TCMs for stroke treatment has increasingly been attracting attention. Celastrol (3-Hydroxy-24-nor-2-oxo-1(10),3,5,7-friedelatetraen-29-oic acid), a pentacyclic triterpene isolated from the traditional Chinese medicine Tripterygium wilfordii Hook. F., with various pharmacological activities including anti-inflammatory [7,8,9], anti-oxidative effects [10,11,12], and more importantly, modulating lipid metabolism [13,14]. In recent years, the effect of celastrol on the CNS has received much attention. Increasing studies found that celastrol is an effective neuroprotective agent against cerebral ischemic injury. A previous study has illustrated that celastrol improved ischemic brain damage in a rat permanent middle cerebral artery occlusion (pMCAO) model via downregulating p-JNK, p-c-Jun and NF-κB [15]. More recently, celastrol has been reported to ameliorate ischemic stroke-induced brain injury by promoting IL-33/ST2-mediated M2 microglial polarization [16]. Another study also demonstrated that celastrol could attenuate neuroinflammation, decrease neuronal apoptosis and oxidative stress through inhibiting HMGB1/NF-κB signaling pathway, thereby alleviating cerebral I/R injury in rats [17]. These results indicated that celastrol may become a promising therapeutic drug for the treatment of ischemic stroke. However, the protective effect and molecular mechanism of celastrol in cerebral I/R injury still need further investigation.

The brain is a highly lipid-dense organ and lipid homeostasis is necessary for maintaining the normal structure and function of the CNS. The changes in lipid metabolism after stroke may aggravate or reduce brain damage, thus affecting the prognosis of stroke. Lipidomics is a high-throughput analytical technology based on liquid chromatography-tandem mass spectrometry (LC-MS/MS), which is a key approach to further understand the pathogenesis of ischemic stroke, and can be used to screen lipid biomarkers with clinical diagnosis, treatment, and prognostic value in various biological samples (serum, plasma, urine, tissues, etc.) [18,19]. Moreover, lipidomics can also help to explore the potential mechanism of drug therapy in ischemic stroke [13,19]. For example, serum levels of high-density lipoprotein cholesterol (HDL-C) were contributed to early neurological deterioration in patients with ischemic stroke [20]. Other research reported that lower serum triglyceride (TG) and HDL-C levels were responsible for the increase in stroke severity [21]. Lipids such as lysophosphatidylcholines (LPCs), phosphatidylcholines (PCs), sphingomyelins (SMs), phosphatidylethanolamines (PEs) and ceramides (CMs) in ischemic brain tissues were reported to be involved in the pathogenesis of ischemic stroke [19,22,23]. Furthermore, regulation of lipid levels and lipid-related metabolic pathways including glycerophospholipid metabolism, arachidonic acid metabolism and sphingolipid metabolism is also an important mechanism for the neuroprotective effects of stroke treatment drugs [24,25,26,27]. These investigations indicated that metabolites of lipid metabolism may play a critical role in cerebral I/R injury rather than merely act as biomarkers. Recently, celastrol has been proved to reverse lipid metabolism disorders in different diseases. Lipidomic analysis showed that celastrol restored the levels of PCs, LPCs, SMs and CMs in 3T3-L1 adipocytes and hyperlipidemia mouse serum by regulating lipid synthesis and metabolism associated genes [13]. Study of experimental colitis found that celastrol played an anti-inflammatory effect by intervening LPC and SM metabolism in serum and colon of colitis mice [14]. However, celastrol on lipid metabolism in the brain of cerebral I/R injury and the relationship between the metabolites of lipid metabolism and neuroprotection remains largely unknown.

Therefore, in this context, we examined the neuroprotection of celastrol in transient middle cerebral artery occlusion (tMCAO) mice model via TTC staining and TUNEL test. Additionally, we then applied lipidomics investigations based on UPLC/MS to evaluate the different lipid metabolites in the ipsilateral hemisphere and plasma in tMCAO mice model, especially the differences between the celastrol treated or not after the onset of reperfusion. Furthermore, we screened the lipid metabolites via bioinformatics and found that sphingolipid and glycerophospholipid metabolic pathways may play an important role during the cerebral I/R injury, which may still need further investigation to mine the underlying mechanism.

## 2. Results

### 2.1. Cerebral Infarction Evaluation and TUNEL Staining

**Figure 1:**
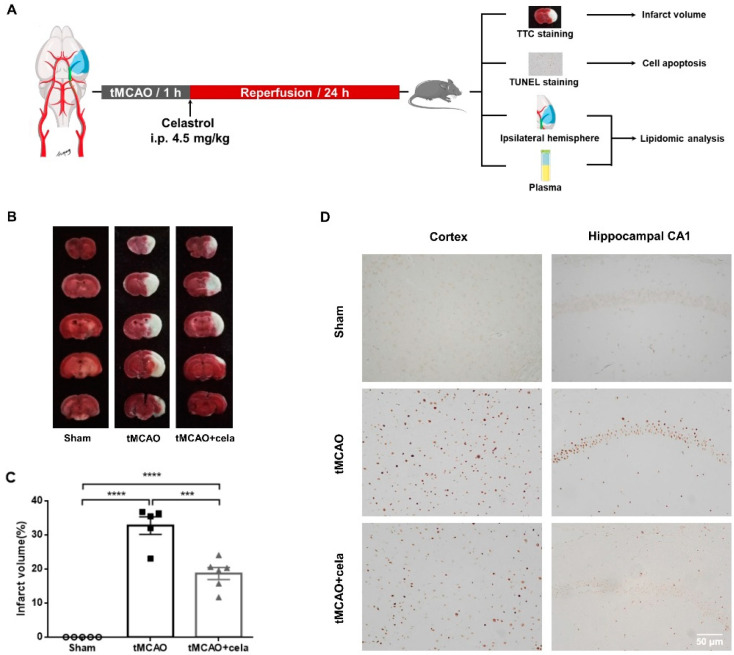
Celastrol effectively protected against cerebral ischemia reperfusion injury in mice. (**A**) Flow chart of experimental protocol. (**B**) Representative TTC staining images at 24 h after reperfusion. The ischemic portion appeared white and the non-ischemic portion appeared red. (**C**) Quantitative analysis of infarct volume (*n* = 5–6). (**D**) Results of TUNEL staining in the ipsilateral cerebral cortex and hippocampal CA1 regions (magnification: 400×, scale bar = 50 μm). Data were presented as mean ± SEM. ^***^ *p* < 0.001, ^****^ *p* < 0.0001.

The tMCAO mice model was successfully built, the mean CBF of MCA was decreased 80%. After 1 h occlusion, the suture was removed to allow the reperfusion. The mean CBF of MCA was increased to 70%. At 24 h after reperfusion, the ischemic hemisphere brain samples and blood samples were collected for further tests (Figure 1A). TTC staining was used to distinguish infarcted brain tissues to assess the severity of brain damage, as shown in results, no cerebral infarct lesion was observed in Sham group, whereas the infarct volume was apparently increased in tMCAO mice (32.7 ± 5.1%) (Figure 1B,C, *p* < 0.0001 vs. Sham group). Treatment with 4.5 mg/kg celastrol significantly reduced I/R-induced infarct volume to 18.7 ± 3.9% (Figure 1B,C, *p* < 0.001 vs. tMCAO group). TUNEL staining was used to examine the anti-apoptotic effects of celastrol. More apoptotic cells were observed in tMCAO group than that of Sham group. While compared with tMCAO group, the apoptotic cells in the cortex and hippocampus CA1 regions were decreased in Celastrol-treated tMCAO group (Figure 1D). These findings suggested that celastrol possessed significant neuroprotective properties against cerebral I/R injury in experimental animals.

### 2.2. Multivariate Data Analysis for Plasma and Brain Samples

**Figure 2:**
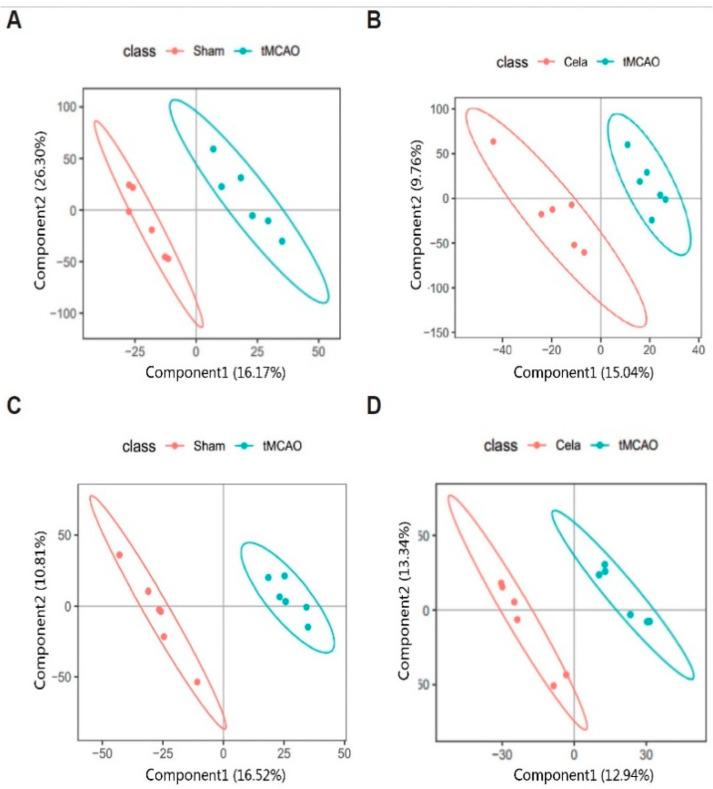
Non-targeted lipidomics analysis of brain and plasma after tMCAO and Cela-treated tMCAO from mice. Plot of brain and plasma PLS-DA between Sham and tMCAO (**A**,**C**) groups, tMCAO and Cela-treated tMCAO (**B**,**D**) groups.

Metabolomics profiling of the plasma and brain samples was carried out by using UPLC/MS. PLS-DA was used to further investigate the degree of similarities and differences between the Sham, tMCAO and Cela-treated tMCAO groups. The results identified a clear separation of the three groups in plasma (Figure 2A,B) and brain (Figure 2C,D). We can clearly see two principal components including PC1 (16.17%) and PC2 (26.30%) of plasma (Figure 2A) and two separate components including PC1 (16.52%) and PC2 (10.81%) of brain (Figure 2C). Similarly, the plasma of Cela-treated and untreated tMCAO mice was also significantly divided into PC1 (15.04%) and PC2 (29.76%) (Figure 2B). Meanwhile, the brain of Cela-treated and untreated tMCAO mice were always separated clearly (Figure 2D). These may indicate a broad role of celastrol in treating tMCAO mice.

### 2.3. Differentially Expressed Lipids (DELs) Identification

**Figure 3:**
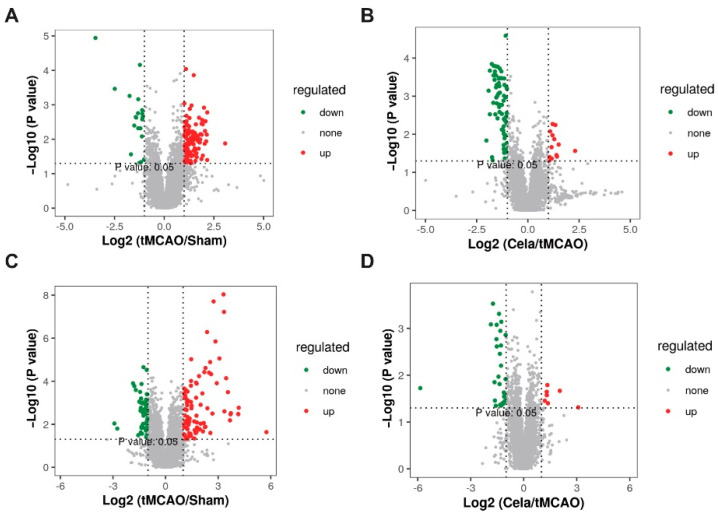
The volcano map showed the differential lipids expression level among the Sham mice, tMCAO mice and Cela-treated tMCAO mice. The red color indicates the upregulated lipids, while green color represents the downregulated lipids. (**A**) plasma_tMCAO/Sham, (**B**) plasma_Cela/tMCAO, (**C**) brain_tMCAO/Sham and (**D**) brain_ Cela/tMCAO groups.

**Figure 4:**
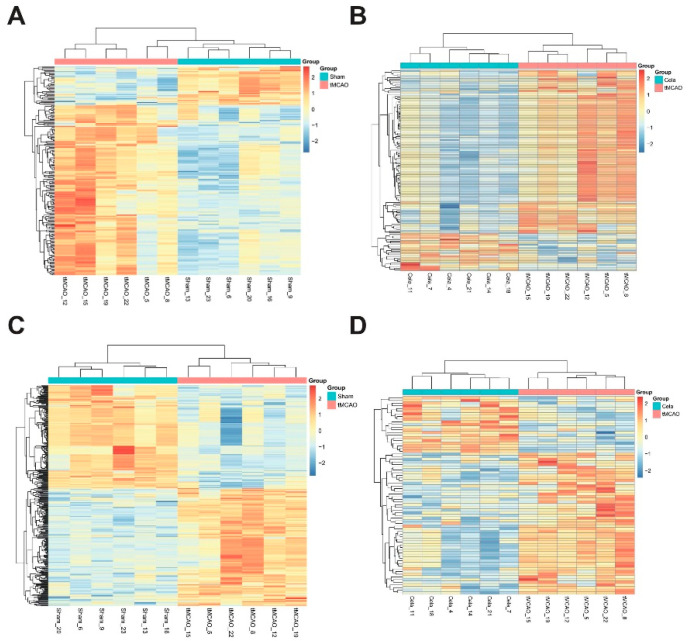
Heatmap of the expression of differential lipids in different comparison groups. (**A**) plasma_tMCAO/Sham, (**B**) plasma_Cela/tMCAO, (**C**) brain_tMCAO/Sham and (**D**) brain_ Cela/tMCAO groups.

The fold change (FC) > 2, *p* < 0.05 and VIP value > 1 were used as the threshold to identify the DELs between two groups. A total of 128 DELs (100 upregulated and 28 downregulated) were found between tMCAO and Sham groups in plasma (Table S1), while there were 54 DELs (14 increased and 40 decreased) after tMCAO mice treated by celastrol in plasma (Table S2). The volcano plots (Figure 3A,B) and heatmap (Figure 4A,B) showed the expression level of DELs between Sham, tMCAO and Celastrol-treated tMCAO groups in plasma. There were 171 DELs (103 increased and 68 decreased) were analyzed between tMCAO and Sham groups in brain (Table S3), otherwise there were 29 DELs (7 increased and 22 decreased) in brain after tMCAO mice treated by celastrol (Table S4). The volcano plots (Figure 3C,D) and heatmap (Figure 4C,D) also showed the expression level of DELs between Sham, tMCAO and Celastrol-treated tMCAO groups in ischemic hemisphere. From the results above, significant differences of endogenous lipid metabolites between Sham, tMCAO and Celastrol-treated tMCAO mice were identified. Therefore, celastrol may play a role in the treatment of tMCAO mice by altering the expression of certain lipid metabolites.

**Figure 5:**
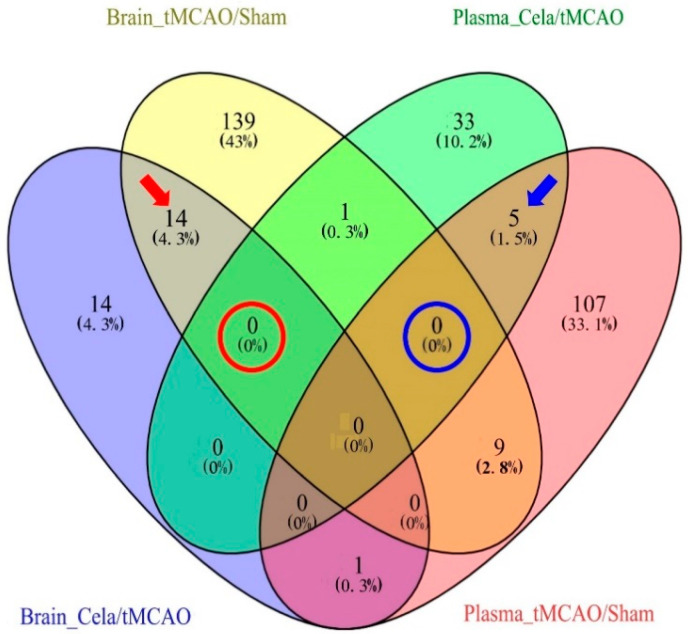
The common and unique differential lipids of different comparison groups.

In order to evaluate the key lipids which induced by stroke and celastrol treatment, we used a venn map to find the common lipids between tMCAO mice and Celastrol-treated tMCAO mice in plasma and brain. In the ischemic hemisphere, the analysis showed that there were 14 common DELs between brain_tMCAO/Sham and brain_Cela/tMCAO groups (Figure 5, Red Arrow). Compared with Sham group, the levels of 13 lipid metabolites (i. 2-hydroxybutyric acid, ii. PS (dime(11,3)/dime(11,5)), iii. PI (40:5), iv. 1,2-di-(9z,12z,15z-octadecatrienoyl)-3-(galactosyl-alpha-1-6-galactosyl-beta-1)-glycerol, v. 4-phenylpyridine, vi. (1aalpha,2beta,3alpha,11calpha)-1a,2,3,11c-tetrahydro-6,11-dimethylbenzo[6,7]phenanthro[3,4-b]oxirene-2,3-diol, vii. erucic acid, viii. CE (20:5), ix. ceramide (d18:1/25:0), x. jubanine c, xi. TG (36:4), xii. PS (dime(11,3)/dime(13,5)), xiii. 3-o-sulfogalactosylceramide (d42:2) were increased in tMCAO group, and only one lipid metabolite (l-aspartyl-4-phosphate) were decreased in tMCAO group. The changes in all of the 14 DELs were reversed when treated by celastrol. Meanwhile, in the plasma, the analysis only exhibited five common DELs between plasma_tMCAO/Sham and plasma_Cela/tMCAO groups. Compared with Sham group, the 4 lipid metabolites (i. PS (42:8), ii. PE (42:7), iii. all trans decaprenyl diphosphate, iv. 3-o-sulfogalactosylceramide) were significantly increased in tMCAO group, and also only one lipid metabolite (PC (44:10) was decreased in tMCAO group (Figure 5, Blue Arrow). All of the five DELs were found to exhibit a normal-level tendency in Celastrol-treated tMCAO mice. Unexpectedly, we did not find common DELs not only between plasma_Cela/tMCAO and brain_Cela/tMCAO groups (Figure 5, Red Circle), but also between plasma_tMCAO/Sham and brain_tMCAO/Sham groups (Figure 5, Blue Circle). These results indicated that the lipid metabolism in the ischemic hemisphere and plasma of cerebral I/R injury, no matter treated by celastrol or not, may share several common metabolic pathways, but the specific metabolic process/pattern may different.

### 2.4. Pathway Analysis and Possible Underlying Pathways

**Figure 6:**
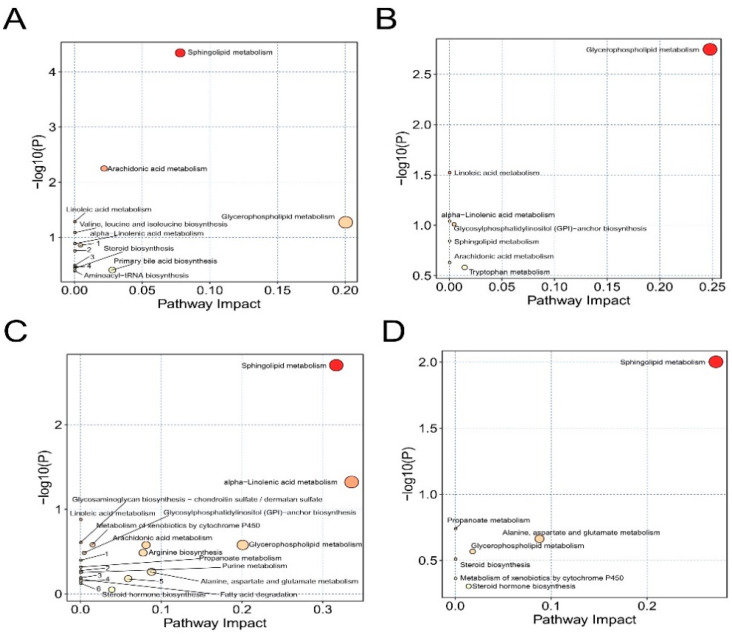
Summary of KEGG pathway analysis with MetaboAnalyst. (**A**) plasma_tMCAO/Sham, (**B**) plasma_Cela/tMCAO, (**C**) brain_tMCAO/Sham and (**D**) brain_Cela/tMCAO.

In order to further explore the effect of celastrol on metabolic pathways in tMCAO mice, the significantly changed lipids were subjected to MetaboAnalyst software and the enriched KEGG pathway between the Sham, tMCAO and Celastrol-treated tMCAO groups were exhibited by bubble plots (Figure 6, Table 1, Table 2, Table 3 and Table 4, Tables S5 and S6). There are nine and seven enriched pathways in plasma_tMCAO/Sham and plasma_Cela/tMCAO groups, respectively (Figure 6A,B). Additionally, six common pathways (i. glycerophospholipid metabolism, ii. linoleic acid metabolism, iii. alpha-linolenic acid metabolism, iv. glycosylphosphatidylinositol (GPI)-anchor biosynthesis, v. sphingolipid metabolism, vi. arachidonic acid metabolism) were found between plasma_tMCAO/Sham and plasma_Cela/tMCAO groups. Meanwhile, there are 14 and 7 enriched pathways in brain_tMCAO/Sham and brain_Cela/tMCAO groups, respectively (Figure 6C,D). A total of seven common pathways (i. sphingolipid metabolism, ii. propanoate metabolism, iii. alanine, aspartate and glutamate metabolism, iv. glycerophospholipid metabolism, v. steroid biosynthesis, vi. metabolism of xenobiotics by cytochrome P450, vii. steroid hormone biosynthesis) were enriched between brain_tMCAO/Sham and brain_Cela/tMCAO groups. Among the above enriched pathways, we found that sphingolipid metabolism and glycerophospholipid metabolism were not only significantly enriched (Table 1, Table 2, Table 3 and Table 4, *p* < 0.05) in most comparison groups, but also significantly enriched (*p* < 0.05) in brain and plasma no matter celastrol treatment or not. Furthermore, three top frequency metabolites including phosphatidylcholine, phosphatidylethanolamine and sulfatide, which belong to glycerophospholipid metabolism and sphingolipid metabolism as well (Table S7), may closely related to the potential mechanism of lipid metabolism as well as the neuroprotective effect of celastrol in cerebral I/R injury.

## 3. Discussion

Lipids are essential for sustaining the normal structure and function of the CNS, among which fatty acids, triglycerides, phospholipids, sterol lipids, and sphingolipids are the most important categories [22]. Previous studies suggested that the curative effect of some natural medicines on ischemic stroke may be associated with its regulation of lipid metabolism such as glycerophospholipid and sphingolipid metabolism [28]. Additionally, celastrol has been clarified to have the neuroprotective effect on ischemic stroke as well as the lipid-regulating effect on diseases such as colitis [15]. Under this background, our study also found that celastrol could alleviate cerebral I/R injury in tMCAO mice, and celastrol exhibited anti-apoptosis effect in our research. Furthermore, our lipidomics results on the ischemic hemisphere and plasma of mice, revealed that the neuroprotection of celastrol may related with its effect by altering the level of sulfatide, phosphatidylcholine (PC), and phosphatidylethanolamine (PE) in sphingolipid and glycerophospholipid metabolism.

In our study, the pathway enrichment analysis showed sphingolipid and glycerophospholipid metabolism were significantly enriched (*p* < 0.05) between the Sham, tMCAO and Celastrol-treated tMCAO groups. Brain contains large proportion of lipids, particularly sphingolipids and glycerophospholipids, which have been regarded as the critical constituent of the cell membrane and participate in maintaining the normal physiological process of CNS [29,30,31,32,33]. Accumulating studies have identified that these two lipid metabolism disorders contributed to the pathogenesis of ischemic stroke [23,34]. Consistently, our lipidomic analysis illustrated the levels of sphingolipids and glycerophospholipids were altered in tMCAO group. Moreover, consistent with the previous studies which highlighted that celastrol was able to interfere with sphingolipid and glycerophospholipid metabolism [13], we observed the alteration of these two lipids induced by tMCAO were blocked after celastrol intervention, suggesting the underlying mechanism of celastrol in relieving cerebral I/R injury was very likely related to its regulation of sphingolipid and glycerophospholipid metabolic pathway.

Among many sphingolipids, our lipidomic analysis found sulfatide was significantly up-regulated in brain and plasma after cerebral I/R injury, which reversed by celastrol, implying that sulfatide may be a target of celastrol in treating cerebral I/R injury. Sulfatide, also known as 3-O-sulfogalactosylceramide, is abundantly present in the myelin sheath of the CNS, plays a critical role in regulating and maintaining myelin-associated physiological events [35,36,37]. To date, several studies have focused on the relationship between sulfatide and CNS diseases [38,39,40]. Sulfatide was reported to induce neuronal apoptosis and increase NF-κB, p-JNK, and p-c-Jun in brain [41]. Intriguingly, celastrol and sulfatide had the opposite effect on the same targets in brain, as celastrol was found to suppress the ischemic stroke-induced upregulation of NF-κB, p-JNK, and p-c-Jun [15,17]. Since NF-κB and JNK pathways act as a key role in regulating ischemia-induced apoptosis [42,43], the relationship between celastrol, sulfatide, and apoptosis attracted our attention. Previous studies have proved that the protective effect of celastrol on ischemic stroke is related to its regulation of apoptotic responses via inhibiting NF-κB [17]. In this study, our results confirmed that celastrol was able to prevent ischemia-induced neuronal apoptosis and regulate sulfatide metabolism. Based on all the above, we hypothesize the mechanism of celastrol protects against cerebral I/R injury may be attributed to the decrease in sulfatide, which consequently inhibits sulfatide-triggered elevated expressions of NF-κB, p-JNK, and p-c-Jun, thus ultimately reduces ischemic stroke-induced cell apoptosis. However, the underlying mechanism still needs further research to explore.

As the main category of glycerophospholipids, the dysregulation of PC and PE metabolism was observed in tMCAO group, indicating that these two lipids may be involved in the pathological development of cerebral I/R injury. PC and PE are found to be deemed as the major phospholipids in cell membranes, which are responsible for protein biogenesis and activation, oxidative phosphorylation, and mitochondrial stability [33,44,45]. As we know, PC and PE can be hydrolyzed by lipoprotein-associated phospholipaseA2 (PLA2) into lysophosphatidylcholines (LPC) and lysophosphatidylethanolamine (LPE), thereby leading to the production of the second messenger arachidonic acid (AA) [27,46]. The release of AA leads to the activation of cyclooxygenase (COX) which could cause cell apoptosis [47]. In addition, it was found that PLA2 was the target of celastrol and inhibition of PLA2 could reduce the hydrolysis of PC and PE, exerting protective effects in ischemic stroke [27,48]. In our study, celastrol was able to attenuate cerebral I/R-induced PC and PE metabolism disturbance. Additionally, PLA2 may be involved in this process. Meanwhile, the anti-apoptotic effect of celastrol on cerebral ischemia was observed in our results. As apoptosis is related with phospholipid metabolism, we supposed that celastrol may regulate PC and PE metabolism by targeting PLA2, and eventually exert anti-apoptotic effect in ischemic brain, but whether our hypothesis is correct still needs experiments to explore and validate.

Taken together, lipidomics based on UPLC/MS was applied in this study to investigate the potential relationship among alteration of lipid metabolites, neuroprotective effect, and mechanisms of celastrol on cerebral I/R injury in tMCAO mice. Our results confirmed that Celastrol-treated tMCAO mice reduced infarct volume and apoptosis-positive cells, which suggested the neuroprotection and anti-apoptosis effect of celastrol. Moreover, lipidomics analysis showed that the mechanism of celastrol in the treatment of cerebral I/R injury might partially associated with its affection on lipid metabolites in sphingolipid and glycerophospholipid metabolism pathways. Among those metabolites, sulfatide, PC, and PE may be the most important targets and the potential mechanism may relate with their anti-apoptosis effect. Although the current study provides new insights into the relationship between regulating lipid metabolism effect and the mechanism of neuroprotection when treated by celastrol, further research is still needed to elucidate the underlying mechanism of lipid metabolites-related neuroprotection in cerebral ischemic-reperfusion injury.

## 4. Materials and Methods

### 4.1. Animals

Male C57BL/6 mice (weighing 18–22 g) were purchased from the Laboratory Animal Center, Chongqing Medical University, China (License number: SYXK(Chongqing)2018-0003). The animals were housed under sterile and controlled conditions (temperature 24 ± 2 °C, relative humidity 60 ± 10%) with a 12 h light/dark rhythm and had free access to standard food and water. All animals adapted to the environment for 5 days before experimentation. All animal experiments were carried out in accordance with the Guide for the Care and Use of Laboratory Animals and were approved by the Animal Care Committee of Chongqing Medical University. Mice were randomly divided into 3 groups: Sham group, tMCAO group, Celastrol-treated tMCAO group. Celastrol (purity 99.56%, Selleckchem, Houston, TX, USA) was dissolved in 1% dimethyl sulfoxide (dimethyl sulfoxide diluting with 0.9% saline solution) at the concentration of 4.5 mg/kg and injected intraperitoneally at the onset of reperfusion. The mice in Sham and tMCAO groups received equal volume of 1% dimethyl sulfoxide. The concentration of celastrol used in this experiment was converted from the concentration reported in previous study.

### 4.2. Transient Middle Cerebral Artery Occlusion (tMCAO) Model

Focal cerebral ischemia was induced using a transient middle cerebral artery occlusion (tMCAO) model according to the previous method [49,50]. In brief, mice were anesthetized with an intraperitoneal injection of 40 mg/kg sodium pentobarbital. A median incision in the neck was made to expose the right common carotid artery (CCA), external carotid artery (ECA) and internal carotid artery (ICA). A 4–0 silicon-coated nylon suture (diameter 0.21–0.25 mm, Yushun Biotech, Pingdingshan, Henan, China) was inserted from the ECA to the ICA and gently advanced forward until it obstructed the MCA. The suture was gently withdrawn 1 h after MCAO to restore blood flow for 24 h. The same surgical procedure was performed in Sham group without occluding the MCA. Body temperature was maintained between 36.5 °C to 37.5 °C throughout the experiment. To determine whether the tMCAO model was induced successfully or not, the microtip of Laser Doppler Flowmetry (Periflux System 5000, Perimed, Järfälla, Sweden) was fixed at 1 mm posterior and 5 mm lateral to the bregma to monitor the cerebral blood flow (CBF) in MCA area. A decrease in CBF ≥ 80% during the 1 h ischemic period and a recovery of CBF ≥ 70% within 10 min were considered as a successful model. After fully waking up from anesthesia, the mice were returned to their home cages.

### 4.3. Cerebral Infarct Volume Measurement

Cerebral ischemia/reperfusion (I/R) induced by tMCAO led to severe brain damage in experimental animals. The 2,3,5-triphenyltetrazolium chloride (TTC) staining was used to assess the cerebral infarct volume. 16 mice were randomly divided into Sham group (*n* = 5), tMCAO group (*n* = 5) and Celastrol-treated tMCAO group (*n* = 6). After 1 h MCAO and 24 h reperfusion, mice were anesthetized with sodium pentobarbital (40 mg/kg, I.P.) and decapitated under deep anesthesia. Immediately, brains were removed and frozen at −20 °C for 30 min, and then coronally cut into 5 slices at a thickness of 2 mm. The slices were incubated in a 2% TTC solution (Servicebio, Wuhan, China) and stained at 37 °C for 30 min, and subsequently fixed in 4% paraformaldehyde overnight. After TTC staining, ischemic portion appeared white and the non-ischemic portion appeared red. The infarct volume was evaluated by Image J software (National Institutes of Health, Bethesda, Maryland, MD, USA) and the following calculation formula was used: (contralateral hemisphere area–ipsilateral non-ischemic hemisphere area)/contralateral hemisphere area × 100%.

### 4.4. TUNEL Staining

TdT-mediated dUTP Nick-End Labeling (TUNEL) staining was performed with Colorimetric TUNEL Apoptosis Assay Kit (Beyotime Biotech, Shanghai, China) following the manufacturer’s instruction. After 24 h of reperfusion, animals in each group (3 mice per group) were anesthetized and slowly perfused with PBS and 4% paraformaldehyde for 15 min, respectively. After that, the brain was taken and fixed in 4% paraformaldehyde for 24 h. Then, the brain tissue was embedded by paraffin, and serial cut into coronal sections (5 μm-thick). To determine apoptotic cells, sections obtained 3–3.5 mm regions of the bregma from each animal were stained with TUNEL and apoptosis positive cells in the parietal cortex and hippocampal CA1 regions appeared dark brown under a light microscopy.

### 4.5. Sample Collection and Preparation

Ischemic hemispheres and plasma from mice in Sham (*n* = 6), tMCAO (*n* = 6) and Celastrol-treated tMCAO (*n* = 6) groups were selected for lipidomic analysis. Briefly, mice were sacrificed 24 h after reperfusion, and the ischemic hemisphere were collected immediately. In addition, the blood was collected in an anticoagulation tube containing EDTA and centrifuged at 1200 g at 4 °C for 10 min to obtain the plasma supernatant. The collected plasma and hemispheres were immediately placed in liquid nitrogen for 40 min and then stored at −80 °C. To extract metabolites, the plasma and ischemic hemisphere samples were thawed on ice, then 20 μL of sample was extracted with 120 μL of precooled 50% methanol, vortexed for 1 min and incubated at room temperature for 10 min, the extraction mixture was then stored overnight at −20 °C. After centrifugation at 4000 g for 20 min, the supernatants were transferred into a new 96-well plate. The samples were stored at −80 °C prior to the UPLC-MS analysis. In addition, pooled QC samples were also prepared by combining 10 μL of each extraction mixture.

### 4.6. Chromatography Analysis

All samples were acquired by the UPLC/MS system followed machine orders. Firstly, all chromatographic separations were performed using an ultra-performance liquid-chromatography (UPLC) system (SCIEX, Framingham, Massachusetts, MA, USA). An ACQUITY UPLC T3 column (100 mm × mm, 1.8 µm, Waters, Milford, Massachusetts, MA, USA) was used for the reversed phase separation. The column oven was maintained at 35 °C. The flow rate was 0.4 mL/min and the mobile phase consisted of solvent A (water, 0.1% formic acid) and solvent B (acetonitrile, 0.1% formic acid). Gradient elution conditions were set as follows: 0–0.5 min, 5% B; 0.5–7 min, 5% to 100% B; 7–8 min, 100% B; 8–8.1 min, 100% to 5% B; 8.1–10 min, 5% B. The injection volume for each sample was 4 μL.

### 4.7. Mass Spectrometry Analysis

The eluent was introduced into the TripleTOF5600plus (SCIEX, Framingham, MA, USA) mass spectrometry (MS) to detect metabolites eluted form the column. The MS was operated using the electrospray positive-ion (ESI^+^) and negative-ion (ESI^-^) modes. The curtain gas was set at 30 PSI, Ion source gas1 was set at 60 PSI, Ion source gas2 was set at 60 PSI, and an interface heater temperature was 65 °C. For positive ion mode, the ionspray voltage floating was set at 5000 V. For negative ion mode, the ionspray voltage floating was set at −4500 V. The mass spectrometry data were acquired in IDA mode. The TOF mass range was from 60 to 1200 Da. The survey scans were acquired in 150 ms and as many as 12 product ion scans were collected if exceeding a threshold of 100 counts per second (counts/s) and with a 1+ charge-state. Total cycle time was fixed to 0.56 s. Four-time bins were summed for each scan at a pulser frequency value of 11 kHz through monitoring of the 40 GHz multichannel TDC detector with four-anode/channel detection. Dynamic exclusion was set for 4 s. During the acquisition, the mass accuracy was calibrated every 20 samples. Furthermore, in order to evaluate the stability of the UPLC/MS during the whole acquisition, a quality control sample (pool of all samples) was acquired after every 10 samples.

### 4.8. Data Processing and Statistical Analysis

The data of cerebral infarct volume was presented as mean ± SEM. The statistical significance between groups were performed by one-way analysis of variance (ANOVA) followed by Tukey’s multiple comparison test, GraphPad Prism 6.01 Software (GraphPad, San Diego, CA, USA) was used in our study. *p* < 0.05 was statistically significant.

The acquired MS data pretreatments including peak picking, peak grouping, retention time correction, second peak grouping, and annotation of isotopes and adducts was performed using XCMS software. UPLC/MS raw data files were converted into mzXML format and then processed by the XCMS, CAMERA and metaX toolbox implemented with the R software. Each ion was identified by combining retention time (RT) and *m/z* data. Intensities of each peaks were recorded and a three-dimensional matrix containing arbitrarily assigned peak indices (retention time-*m/z* pairs), sample names (observations) and ion intensity information (variables) was generated.

**Figure 7:**
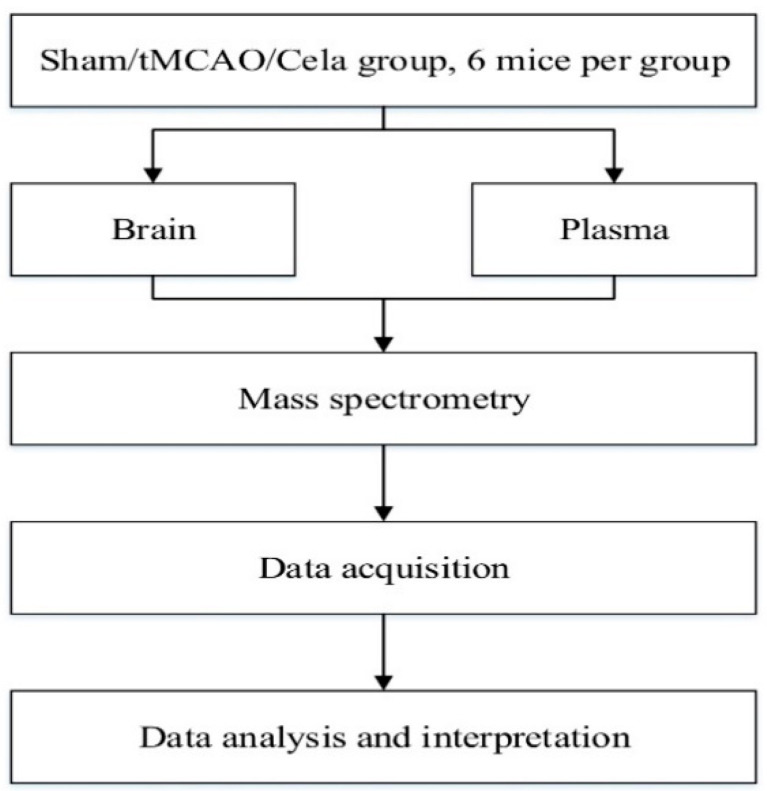
Workflow of the lipidomics.

The KEGG and HMDB databases were used to annotate the metabolites by matching the exact molecular mass data (*m/z*) of samples with those from database. If a mass difference between observed and the database value was less than 10 ppm, the metabolite would be annotated and the molecular formula of metabolites would further be identified and validated by the isotopic distribution measurements. We also used an in-house fragment spectrum library of metabolites to validate the metabolite identification. The workflow was shown in Figure 7.

The intensity of peak data was further preprocessed by metaX. Those features that were detected in less than 50% of QC samples or 80% of biological samples were removed, the remaining peaks with missing values were imputed with the k-nearest neighbor algorithm to further improve the data quality. Partial least-squares-discriminant analysis (PLS-DA) was performed for outlier detection and batch effects evaluation using the pre-processed dataset. Quality control-based robust LOESS signal correction was fitted to the QC data with respect to the order of injection to minimize signal intensity drift over time. In addition, the relative standard deviations of the metabolic features were calculated across all QC samples, and those >30% were then removed.

Student *t*-tests were conducted to detect differences in metabolite concentrations between 2 phenotypes. The *p* value was adjusted for multiple tests using an FDR (Benjamini–Hochberg). Supervised PLS-DA was conducted through metaX to discriminate the different variables between groups. The VIP value was calculated. A VIP cut-off value of 1.0 was used to select important features.

## Figures and Tables

**Table 1:** The enriched KEGG pathway of differential metabolites of tMCAO/Sham in mouse plasma.

Pathway Name	*p*-Value	Metabolite	Impact
Sphingolipid metabolism	0.00005	Sphingomyelin; Digalactosylceramide; Glucosylceramide; Sulfatide	0.07708
Arachidonic acid metabolism	0.00568	5,6-EET; Phosphatidylcholine; 14,15-DHET	0.02120
Linoleic acid metabolism	0.05207	Phosphatidylcholine	0.00000
Glycerophospholipid metabolism	0.05404	Phosphatidylethanolamine; Phosphatidylcholine	0.19895
Valine, leucine and isoleucine biosynthesis	0.08208	L-Isoleucine	0.00000
alpha-Linolenic acid metabolism	0.13013	Phosphatidylcholine	0.00000
Glycosylphosphatidylinositol (GPI)-anchor biosynthesis	0.13945	Phosphatidylethanolamine	0.00399
Terpenoid backbone biosynthesis	0.17581	Dimethylallyl diphosphate	0.00000
Biosynthesis of unsaturated fatty acids	0.32232	(4Z,7Z,10Z,13Z,16Z,19Z)-Docosahexaenoic acid	0.00000
Valine, leucine and isoleucine degradation	0.35138	L-Isoleucine	0.00000
Steroid biosynthesis	0.36546	Cholesterol ester	0.00000

**Table 2:** The enriched KEGG pathway of differential metabolites of Cela/tMCAO in mouse plasma.

Pathway Name	*p*-Value	Metabolite	Impact
Glycerophospholipid metabolism	0.00180	Phosphatidylethanolamine; Phosphatidylcholine; Phosphatidylserine	0.24596
Linoleic acid metabolism	0.03000	Phosphatidylcholine	0.00000
alpha-Linolenic acid metabolism	0.09125	Phosphatidylcholine	0.00000
Glycosylphosphatidylinositol (GPI)-anchor biosynthesis	0.09795	Phosphatidylethanolamine	0.00399
Sphingolipid metabolism	0.14357	Sulfatide	0.00000
Arachidonic acid metabolism	0.23436	Phosphatidylcholine	0.00000
Tryptophan metabolism	0.26262	5-Hydroxykynurenamine	0.01390

**Table 3:** The enriched KEGG pathway of differential metabolites of tMCAO/Sham in mouse brain.

Pathway Name	*p*-Value	Metabolite	Impact
Sphingolipid metabolism	0.00200	Sphingosine; N-Acylsphingosine; Lactosylceramide; Sulfatide	0.31440
alpha-Linolenic acid metabolism	0.04800	Phosphatidylcholine; (9Z,12Z,15Z)-Octadecatrienoic acid	0.33333
Linoleic acid metabolism	0.13204	Phosphatidylcholine	0.00000
Glycosaminoglycan biosynthesis-chondroitin sulfate/dermatan sulfate	0.24701	Chondroitin	0.00000
Metabolism of xenobiotics by cytochrome P450	0.26300	2-Bromoacetaldehyde	0.01418
Arachidonic acid metabolism	0.26558	S-(1,2-Dichlorovinyl)glutathione	0.07980
Glycerophospholipid metabolism	0.26558	(1aalpha,2beta,3alpha,11calpha)-1a,2,3,11c-Tetrahydro-6,11-dimethylbenzo[6,7]phenanthro[3,4-b]oxirene-2,3-diol	0.19895
Glycosylphosphatidylinositol (GPI)-anchor biosynthesis	0.32816	Phosphatidylethanolamine	0.00399
Arginine biosynthesis	0.32816	L-Arginine	0.07614
Terpenoid backbone biosynthesis	0.40074	Dimethylallyl diphosphate	0.00000
Propanoate metabolism	0.48077	2-Hydroxybutanoic acid	0.00000
Drug metabolism-cytochrome P450	0.53720	Alcophosphamide	0.00000
Alanine, aspartate and glutamate metabolism	0.55034	N-Acetyl-L-aspartate	0.08654
Purine metabolism	0.55802	Allantoate;(S)-Ureidoglycolate	0.00012
Biosynthesis of unsaturated fatty acids	0.64315	(9Z,12Z,15Z)-Octadecatrienoic acid	0.00000
Arginine and proline metabolism	0.66326	L-Arginine	0.05786
Fatty acid degradation	0.67289	L-Palmitoylcarnitine	0.00000
Steroid biosynthesis	0.70021	Cholesterol ester	0.00000
Aminoacyl-tRNA biosynthesis	0.74833	L-Arginine	0.00000
Steroid hormone biosynthesis	0.89308	Pregnenolone	0.03776

**Table 4:** The enriched KEGG pathway of differential metabolites of Cela/tMCAO in mouse brain.

Pathway Name	*p*-Value	Metabolite	Impact
Sphingolipid metabolism	0.01000	N-Acylsphingosine; Sulfatide	0.26978
Propanoate metabolism	0.18199	2-Hydroxybutanoic acid	0.00000
Alanine, aspartate and glutamate metabolism	0.21727	N-Acetyl-L-aspartate	0.08654
Glycerophospholipid metabolism	0.27080	1-Acyl-sn-glycero-3-phosphocholine	0.01736
Steroid biosynthesis	0.30871	Cholesterol ester	0.00000
Metabolism of xenobiotics by cytochrome P450	0.43269	(1aalpha,2beta,3alpha,11calpha)-1a,2,3,11c-Tetrahydro-6,11-dimethylbenzo[6,7]phenanthro[3,4-b]oxirene-2,3-diol	0.00000
Steroid hormone biosynthesis	0.49595	11-Deoxycortisol	0.01317

## Data Availability

The data presented in this study are available in Supplementary Materials.

## Supplementary Materials

The following are available online, Table S1: Differential lipid species tMCAO/Sham in mouse plasma, according to significance evaluation and VIP values, Table S2: Table S2 Differential lipid species Cela/tMCAO in mouse plasma, according to significance evaluation and VIP values, Table S3: Differential lipid species tMCAO/Sham in mouse brain, according to significance evaluation and VIP values, Table S4: Differential lipid species Cela/tMCAO in mouse brain, according to significance evaluation and VIP values, Table S5: The enriched KEGG pathway of differential metabolites of Cela/Sham in mouse plasma, Table S6: The enriched KEGG pathway of differential metabolites of Cela/Sham in mouse brain and Table S7: Frequency of lipid metabolites and the involved KEGG pathway.

## Sample Availability

Celastrol is available from Selleck (America). Product No S1290.
