# Supplementary material for: Lipidomic Profiling of Ipsilateral Brain and Plasma after Celastrol Post-Treatment in Transient Middle Cerebral Artery Occlusion Mice Model
Source: Molecules. 2021 Jul 7;26(14):4124. doi: 10.3390/molecules26144124 (PMC8306490; doi:10.3390/molecules26144124)
Supplement: Supplementary file 1 [file molecules-26-04124-s001.zip › molecules-1256265-supplementary.pdf]

Table S1 Differential lipid species tMCAO/Sham in mouse plasma, according to significance evaluation and VIP values

ID	Fold change	p.value	VIP	Regulated	Metabolite	Ion mode
M130T32	2.198	0.008	3.127	up	L-Isoleucine	-
M185T29	4.731	0.030	3.248	up	Phosphodimethylethanolamine	-
M185T32	2.454	0.018	2.887	up	N-[4'-hydroxy-(E)-cinnamoyl]-L-aspartic acid	-
M223T124	2.324	0.037	2.695	up	5,8-Tetradecadienoic acid	-
M239T77	2.956	0.011	2.890	up	L-Menthyl acetoacetate	-
M249T137	2.136	0.029	2.722	up	Norambreinolide	-
M251T152	2.226	0.038	2.917	up	7Z,10Z-Hexadecadienoic acid	-
M275T149	2.087	0.040	1.808	up	19-Norandrosterone	-
M319T122	4.609	0.019	3.691	up	5,6-Epoxy-8,11,14-eicosatrienoic acid	-
M327T172	2.332	0.027	2.991	up	Docosahexaenoic acid	-
M327T32_2	2.064	0.012	2.641	up	4-{1-hydroxy-3-[4-hydroxy-3-(3-methylbut-2-en-1-yl)phenyl]propyl}benzene-1,3-diol	-
M343T118	4.668	0.049	3.224	up	Medroxyprogesterone	-
M403T295	0.076	0.000	6.127	down	3a,7a-Dihydroxy-5b-cholestane	-
M425T201	0.448	0.013	2.566	down	11,13-Dihydrotaraxinic acid glucosyl ester	-
M492T574	0.481	0.030	2.498	down	Ebrotidine	-
M524T160	2.019	0.034	2.468	up	LysoPE(0:0/22:6(4Z,7Z,10Z,13Z,16Z,19Z))	-
M537T197	2.056	0.043	2.332	up	FAHFA(16:0/9-O-18:0)	-
M699T320	0.499	0.003	2.879	down	PE(16:1(9Z)/P-18:1(11Z))	-
M725T380	0.470	0.000	3.136	down	TG(20:0/a-13:0/8:0)	-
M729T345	0.435	0.001	3.071	down	PC(15:0/P-18:1(11Z))	-
M737T210	4.830	0.008	3.936	up	PG(16:0/16:1(9Z))	-
M738T210	4.303	0.010	3.740	up	PE(18:4(6Z,9Z,12Z,15Z)/P-18:1(11Z))	-

M739T210	3.985	0.009	3.722	up	PG(16:0/16:0)	-
M763T304	3.696	0.024	3.333	up	PE(16:0/22:6(4Z,7Z,10Z,13Z,16Z,19Z))	-
M764T212	2.189	0.007	2.451	up	PE(O-16:1(1Z)/22:6(4Z,7Z,10Z,13Z,16Z,19Z))	-
M777T315	2.227	0.008	3.038	up	PC(14:0/22:6(4Z,7Z,10Z,13Z,16Z,19Z))	-
M786T278	2.160	0.014	2.498	up	PC(15:0/20:3(5Z,8Z,11Z))	-
M789T308	2.548	0.009	3.029	up	PE(18:1(11Z)/22:6(4Z,7Z,10Z,13Z,16Z,19Z))	-
M791T327	4.096	0.012	3.665	up	PC(15:0/22:6(4Z,7Z,10Z,13Z,16Z,19Z))	-
M795T325	2.187	0.005	2.827	up	PC(15:0/22:4(7Z,10Z,13Z,16Z))	-
M814T565	0.423	0.013	3.199	down	TG(14:0/15:0/20:3(5Z,8Z,11Z))	-
M818T335	3.541	0.025	2.907	up	3-Decaprenyl-4-hydroxybenzoic acid	-
M819T210	2.888	0.002	3.550	up	PC(18:4(6Z,9Z,12Z,15Z)/P-16:0)	-
M825T558	0.496	0.027	3.042	down	PC(22:1(13Z)/P-18:1(11Z))	-
M830T304	2.442	0.026	2.748	up	PS(18:2(9Z,12Z)/22:6(4Z,7Z,10Z,13Z,16Z,19Z))	-
M831T269	2.519	0.021	3.037	up	PG(18:0/18:0)	-
M833T304	2.351	0.016	2.994	up	Momorcharaside A	-
M834T277	2.811	0.003	3.316	up	PE(20:1(11Z)/22:6(4Z,7Z,10Z,13Z,16Z,19Z))	-
M841T290	0.433	0.001	2.779	down	PS(18:1(11Z)/22:2(13Z,16Z))	-
M845T304	2.785	0.025	2.997	up	Vinaginsenoside R2	-
M846T304	2.502	0.031	2.803	up	PS(18:3(9Z,12Z,15Z)/22:6(4Z,7Z,10Z,13Z,16Z,19Z))	-
M850T478	0.402	0.003	3.074	down	3-demethylubiquinol-10	-
M859T327	2.451	0.023	2.915	up	PS(20:3(5Z,8Z,11Z)/22:5(4Z,7Z,10Z,13Z,16Z))	-
M864T301	2.828	0.012	2.683	up	PE(20:5(5Z,8Z,11Z,14Z,17Z)/24:1(15Z))	-
M874T327	2.395	0.045	2.508	up	PS(20:3(8Z,11Z,14Z)/22:6(4Z,7Z,10Z,13Z,16Z,19Z))	-
M893T322	2.101	0.005	2.846	up	PE(DiMe(11,5)/MonoMe(13,5))	-
M894T322	2.056	0.004	2.815	up	PC(DiMe(11,3)/DiMe(11,5))	-

M285T31	0.419	0.049	2.989	down	Dimethylallylpyrophosphate	+
M286T36	0.091	0.000	5.871	down	2-[(7-hydroxy-1-oxo-1H-isochromen-3-yl)formamido]acetic acid	+
M328T74	2.288	0.043	2.267	up	(9E)-10-nitrooctadecenoic Acid	+
M332T563	0.497	0.038	2.399	down	2-Undecyl-4(1H)-quinolinone N-oxide	+
M346T170	2.005	0.041	2.791	up	Eicosapentaenoyl Ethanolamide	+
M356T108	2.105	0.031	2.356	up	14,15-DiHETrE	+
M370T122	2.917	0.007	3.191	up	O-(2-tetradecenoyl)carnitine	+
M398T145	2.699	0.009	2.916	up	trans-Hexadec-2-enoyl carnitine	+
M401T164	3.055	0.010	3.149	up	7-Ketocholesterol	+
M426T170	4.488	0.002	4.030	up	Oleoylcarnitine	+
M427T170	3.832	0.004	3.745	up	4alpha-Formyl-4beta-methyl-5alpha-cholesta-8,24-dien-3beta-ol	+
M475T238_2	0.446	0.046	2.291	down	Tyromycic acid	+
M480T210	3.268	0.012	3.406	up	PC-M5'	+
M526T160	2.057	0.033	2.568	up	LysoPE(0:0/22:6(4Z,7Z,10Z,13Z,16Z,19Z))	+
M527T160	2.127	0.009	2.815	up	Desmosine	+
M528T160	2.204	0.027	2.803	up	CGP71422	+
M598T220	0.458	0.008	3.338	down	beta-Doradecin	+
M625T307	4.317	0.011	3.828	up	FAHFA(22:6(4Z,7Z,10Z,13Z,16Z,19Z)/9-O-18:2(10E,12Z))	+
M683T340	2.031	0.008	2.727	up	DG(18:2(9Z,12Z)/22:6(4Z,7Z,10Z,13Z,16Z,19Z)/0:0)	+
M701T320	0.299	0.001	4.093	down	PE(16:1(9Z)/P-18:1(11Z))	+
M702T320	0.427	0.000	3.066	down	CE(DiMe(9,3))	+
M703T322	0.404	0.001	2.932	down	PC(18:0/22:6(4Z,7Z,10Z,13Z,16Z,19Z))	+
M718T300	2.514	0.049	2.615	up	CE(20:1(11Z))	+
M727T322	0.403	0.005	3.076	down	PE(18:2(9Z,12Z)/P-18:1(11Z))	+
M728T277	2.779	0.014	2.988	up	N-hexadecanoylsphinganine-1-phosphocholine	+

M728T323	0.370	0.002	3.351	down	CE(11:1D3)	+
M729T273	2.143	0.008	2.607	up	PC(14:0/18:3(6Z,9Z,12Z))	+
M729T278_1	2.798	0.021	2.924	up	SM(d18:0/16:0)	+
M729T321	2.199	0.004	2.737	up	CerP(d18:1/24:1(15Z))	+
M731T345	0.463	0.001	2.952	down	PC(15:0/P-18:1(11Z))	+
M732T311	2.552	0.049	2.504	up	Ubiquinol 8	+
M732T345	0.471	0.002	2.909	down	PC(14:1(9Z)/P-18:1(11Z))	+
M733T311	2.287	0.046	2.408	up	SM(d18:1/18:0)	+
M734T311	2.228	0.046	2.426	up	DG(i-16:0/a-25:0/0:0)	+
M739T210	3.546	0.007	3.719	up	Glucosylceramide (d18:1/16:0)	+
M740T210	3.012	0.004	3.557	up	SM(d18:0/16:1(9Z)(OH))	+
M743T316	0.352	0.004	3.631	down	PC(16:1(9Z)/P-18:1(11Z))	+
M744T316	0.376	0.002	3.522	down	CE(MonoMe(11,5))	+
M756T210	4.152	0.006	3.895	up	PA(18:1(11Z)/22:2(13Z,16Z))	+
M756T299	8.372	0.013	4.058	up	SM(d18:0/20:2(11Z,14Z))	+
M760T334	3.844	0.030	3.025	up	SM(d18:1/20:0)	+
M761T210	3.197	0.010	3.455	up	PS(16:0/18:2(9Z,12Z))	+
M765T304	2.967	0.020	3.211	up	PE(16:0/22:6(4Z,7Z,10Z,13Z,16Z,19Z))	+
M766T304	2.911	0.018	3.202	up	PE-NMe(14:1(9Z)/22:6(4Z,7Z,10Z,13Z,16Z,19Z))	+
M769T329	2.226	0.034	2.545	up	PC(15:0/20:4(5Z,8Z,11Z,14Z))	+
M773T320	0.401	0.002	3.191	down	PE(20:4(5Z,8Z,11Z,14Z)/P-18:1(11Z))	+
M782T210	2.796	0.000	3.686	up	PS(16:1(9Z)/20:4(5Z,8Z,11Z,14Z))	+
M783T290	0.464	0.003	2.657	down	PC(14:0/22:4(7Z,10Z,13Z,16Z))	+
M784T226	2.045	0.020	2.470	up	PA(18:2(9Z,12Z)/24:1(15Z))	+
M785T287	2.427	0.014	3.021	up	PS(16:0/20:4(5Z,8Z,11Z,14Z))	+

M785T290	0.178	0.000	4.436	down	PC(14:0/20:0)	+
M786T288	2.148	0.022	2.647	up	PE(DiMe(9,3)/DiMe(9,3))	+
M787T304	2.312	0.030	2.714	up	PE(18:3(6Z,9Z,12Z)/22:6(4Z,7Z,10Z,13Z,16Z,19Z))	+
M788T304	2.701	0.006	3.412	up	Narasin	+
M789T293	2.061	0.046	2.479	up	PE(18:2(9Z,12Z)/22:6(4Z,7Z,10Z,13Z,16Z,19Z))	+
M791T308	3.131	0.009	3.418	up	PE(18:1(11Z)/22:6(4Z,7Z,10Z,13Z,16Z,19Z))	+
M792T308	2.872	0.007	3.344	up	PC(18:4(6Z,9Z,12Z,15Z)/18:4(6Z,9Z,12Z,15Z))	+
M793T327	3.322	0.019	3.331	up	PC(15:0/22:6(4Z,7Z,10Z,13Z,16Z,19Z))	+
M807T299	2.199	0.016	2.754	up	PC(16:0/22:6(4Z,7Z,10Z,13Z,16Z,19Z))	+
M811T325	2.086	0.006	2.735	up	PC(16:0/22:4(7Z,10Z,13Z,16Z))	+
M812T305	2.044	0.044	2.201	up	PC(15:0/22:5(4Z,7Z,10Z,13Z,16Z))	+
M813T308	2.498	0.042	2.731	up	PE(20:4(5Z,8Z,11Z,14Z)/22:6(4Z,7Z,10Z,13Z,16Z,19Z))	+
M815T327	2.895	0.009	3.206	up	PE(20:3(5Z,8Z,11Z)/22:6(4Z,7Z,10Z,13Z,16Z,19Z))	+
M816T327	3.675	0.004	3.801	up	PE-NMe(18:4(6Z,9Z,12Z,15Z)/22:6(4Z,7Z,10Z,13Z,16Z,19Z))	+
M822T310	2.263	0.019	2.748	up	PC(16:1(9Z)/22:6(4Z,7Z,10Z,13Z,16Z,19Z))	+
M827T329	2.213	0.043	2.542	up	PE(22:0/P-18:0)	+
M837T322	2.391	0.010	3.102	up	PC(18:0/22:5(4Z,7Z,10Z,13Z,16Z))	+
M837T327	3.513	0.010	3.968	up	PE(22:6(4Z,7Z,10Z,13Z,16Z,19Z)/22:6(4Z,7Z,10Z,13Z,16Z,19Z))	+
M838T323	2.737	0.029	3.018	up	PE(20:0/22:6(4Z,7Z,10Z,13Z,16Z,19Z))	+
M844T310	2.020	0.001	3.067	up	PC(18:4(6Z,9Z,12Z,15Z)/22:6(4Z,7Z,10Z,13Z,16Z,19Z))	+
M859T322	2.326	0.007	3.091	up	PC(20:2(11Z,14Z)/22:6(4Z,7Z,10Z,13Z,16Z,19Z))	+
M861T288	2.026	0.022	2.791	up	PG(18:0/22:6(4Z,7Z,10Z,13Z,16Z,19Z))	+
M865T299	2.055	0.024	2.602	up	3-O-Sulfogalactosylceramide (d18:1/22:0)	+
M866T299	2.063	0.017	2.692	up	All trans decaprenyl diphosphate	+
M883T275	0.436	0.005	2.989	down	PC(22:4(7Z,10Z,13Z,16Z)/22:6(4Z,7Z,10Z,13Z,16Z,19Z))	+

M893T323	2.532	0.009	3.156	up	3-O-Sulfogalactosylceramide (d18:1/24:0)	+
M894T323	2.730	0.008	3.290	up	TG(18:3(6Z,9Z,12Z)/18:4(6Z,9Z,12Z,15Z)/18:3(6Z,9Z,12Z))	+
M906T367	2.559	0.010	3.313	up	Galabiosylceramide (d18:1/9Z-18:1)	+
M912T299	2.025	0.008	2.657	up	PI(18:0/22:6(4Z,7Z,10Z,13Z,16Z,19Z))	+
M920T323	2.398	0.023	2.953	up	TG(18:4(6Z,9Z,12Z,15Z)/22:6(4Z,7Z,10Z,13Z,16Z,19Z)/18:4(6Z,9Z,12Z,15Z))	+
M921T323	3.364	0.002	3.675	up	PC(o-22:0/22:3(10Z,13Z,16Z))	+

Table S2 Differential lipid species Cela/tMCAO in mouse plasma, according to significance evaluation and VIP values

ID	Fold change	p.value	VIP	Regulated	Metabolite	Ion mode
M161T28	0.393	0.007	3.273	down	2-Methyl-1-methylthio-2-butene	-
M179T29	0.295	0.003	3.902	down	5-Hydroxykynurenamine	-
M179T32	0.351	0.001	3.910	down	Purine	-
M243T29	2.066	0.041	2.547	up	Nopalinic acid	-
M299T136	2.333	0.025	3.069	up	(R)-3-Hydroxy-Octadecanoic acid	-
M326T28	0.290	0.043	3.377	down	Diloxanide	-
M426T564	2.125	0.023	3.083	up	Trospium chloride	-
M436T184	2.317	0.001	3.379	up	PE(P-16:0e/0:0)	-
M517T251	3.023	0.048	3.272	up	Blumenol C O-[rhamnosyl-(1->6)-glucoside]	-
M858T256	0.329	0.001	4.189	down	PI(16:0/20:4(5Z,8Z,11Z,14Z))	-
M859T256	0.344	0.001	4.055	down	PS(20:3(5Z,8Z,11Z)/22:5(4Z,7Z,10Z,13Z,16Z))	-
M860T260	0.481	0.014	2.877	down	PI(16:0/20:3(5Z,8Z,11Z))	-
M864T301	2.156	0.014	2.802	up	PE(20:5(5Z,8Z,11Z,14Z,17Z)/24:1(15Z))	-
M866T314	2.144	0.034	3.338	up	PE(20:4(5Z,8Z,11Z,14Z)/24:1(15Z))	-

M867T305	0.450	0.026	3.554	down	PS(18:3(6Z,9Z,12Z)/24:1(15Z))	-
M872T265	0.459	0.003	3.494	down	Azaspiracid 2	-
M884T260	0.404	0.008	3.529	down	PI(16:0/22:5(4Z,7Z,10Z,13Z,16Z))	-
M885T260	0.401	0.003	3.763	down	PS(22:4(7Z,10Z,13Z,16Z)/22:5(4Z,7Z,10Z,13Z,16Z))	-
M886T259	0.422	0.006	3.420	down	PI(16:0/22:4(10Z,13Z,16Z,19Z))	-
M887T276	0.307	0.000	4.641	down	PS(22:2(13Z,16Z)/22:6(4Z,7Z,10Z,13Z,16Z,19Z))	-
M888T276	0.351	0.000	4.270	down	PI(16:0/22:3(10Z,13Z,16Z))	-
M889T287	0.460	0.005	3.245	down	PS(22:2(13Z,16Z)/22:5(4Z,7Z,10Z,13Z,16Z))	-
M935T299	0.426	0.013	3.061	down	PS(DiMe(11,5)/MonoMe(13,5))	-
M377T569	2.228	0.022	2.824	up	MG(0:0/18:2(9Z,12Z)/0:0)	+
M426T61	4.941	0.027	3.947	up	3a,7a,12b-Trihydroxy-5b-cholanoic acid	+
M430T167	2.296	0.011	2.711	up	5-(2,3-Dihydroxy-3-methylbutyl)-4-(3,4-epoxy-4-methylpentanoyl)-3,4-dihydroxy-2-isopentanoyl-2-cyclopenten-1-one	+
M435T568	2.835	0.019	3.102	up	MG(0:0/22:1(13Z)/0:0)	+
M519T567	0.485	0.005	2.574	down	{[4-(5,7-dihydroxy-4-oxo-3,4-dihydro-2H-1-benzopyran-2-yl)-5-hydroxy-13,13-dimethyl-8-oxatetracyclo[7.4.1.0tetradeca-2(7),3,5-trien-9-yl]methoxy}sulfonic acid	+
M600T256	0.341	0.001	3.982	down	Cohibin C	+
M610T569	2.311	0.005	3.188	up	3,4,5-trihydroxy-6-({5-hydroxy-4-oxo-2-phenyl-6-[3,4,5-trihydroxy-6-(hydroxymethyl)oxan-2-yl]-4H-chromen-7-yl}oxy)oxane-2-carboxylic acid	+
M648T573	2.242	0.043	2.520	up	PE(14:1(9Z)/15:0)	+
M668T276	0.408	0.000	3.819	down	DG(18:1(11Z)/22:6(4Z,7Z,10Z,13Z,16Z,19Z)/0:0)	+
M836T303	0.481	0.029	3.071	down	PE(20:1(11Z)/22:6(4Z,7Z,10Z,13Z,16Z,19Z))	+
M861T256	0.378	0.002	3.647	down	PS(18:0/22:5(7Z,10Z,13Z,16Z,19Z))	+
M877T256	0.344	0.001	3.894	down	PC(20:4(5Z,8Z,11Z,14Z)/22:6(4Z,7Z,10Z,13Z,16Z,19Z))	+
M878T256	0.356	0.001	3.858	down	PS(20:3(5Z,8Z,11Z)/22:5(4Z,7Z,10Z,13Z,16Z))	+

M879T260	0.440	0.024	2.644	down	PC(22:6(4Z,7Z,10Z,13Z,16Z,19Z)/22:6(4Z,7Z,10Z,13Z,16Z,19Z))	+
M882T508	2.533	0.006	3.110	up	PA(22:0/a-25:0)	+
M890T276	0.381	0.001	3.744	down	PI(16:0/20:0)	+
M903T256	0.388	0.001	3.672	down	Asparagoside D	+
M903T260	0.358	0.004	3.854	down	PC(22:5(4Z,7Z,10Z,13Z,16Z)/22:6(4Z,7Z,10Z,13Z,16Z,19Z))	+
M904T256	0.439	0.001	3.533	down	PIP(16:1(9Z)/16:1(9Z))	+
M904T260	0.414	0.008	3.382	down	All trans decaprenyl diphosphate	+
M905T259	0.457	0.011	3.019	down	PC(22:4(7Z,10Z,13Z,16Z)/22:6(4Z,7Z,10Z,13Z,16Z,19Z))	+
M907T276	0.328	0.000	4.194	down	PC(22:4(7Z,10Z,13Z,16Z)/22:5(4Z,7Z,10Z,13Z,16Z))	+
M909T260	0.415	0.003	3.431	down	Chlorophyll b	+
M909T282	0.488	0.003	3.045	down	PC(22:2(13Z,16Z)/22:6(4Z,7Z,10Z,13Z,16Z,19Z))	+
M930T260	0.464	0.010	3.088	down	Araliasaponin I	+
M931T313	0.348	0.001	4.088	down	3-O-Sulfogalactosylceramide (d18:1/24:0)	+
M932T276	0.386	0.000	3.973	down	Hoduloside VII	+
M933T276	0.412	0.000	3.809	down	PIP(16:0/18:2(9Z,12Z))	+
M934T275	0.475	0.001	3.335	down	Notoginsenoside R1	+
M988T256	0.452	0.001	3.391	down	Basellasaponin A	+
M989T276	0.493	0.001	3.366	down	Phytolaccasaponin B	+

Table S3 Differential lipid species tMCAO/Sham in mouse brain, according to significance evaluation and VIP values

ID	Fold change	p.value	VIP	Regulated	Metabolite	Ion mode
M103T33	3.075	0.002	3.239	up	2-Hydroxybutyric acid	-
M134T33	0.485	0.033	2.646	down	D-erythro-L-galacto-Nonulose	-

M147T29_1	3.720	0.001	3.671	up	3-(4-hydroxy-3,5-dimethoxyphenyl)prop-2-enoic acid	-
M171T30	3.002	0.001	3.352	up	Ribothymidine	-
M173T35	2.093	0.040	2.705	up	L-Arginine	-
M174T26	0.196	0.000	4.033	down	N-Acetyl-L-aspartic acid	-
M174T28	0.287	0.011	3.977	down	2-ethyl-1,5-dimethyl-3,3-diphenylpyrrolinium (EDDP)	-
M174T37	0.086	0.009	3.931	down	D-Erythroascorbic acid 1'-a-D-xylopyranoside	-
M175T28	0.450	0.029	2.860	down	Allantoic acid	-
M182T29_1	2.024	0.000	2.726	up	Saccharin	-
M190T28	2.694	0.000	3.293	up	Brassilexin	-
M195T29	2.729	0.002	2.680	up	5-(4,7-dihydroxy-6-methoxy-3,4-dihydro-2H-1-benzopyran-3-yl)benzene-1,2,4-triol	-
M213T30	2.024	0.016	2.341	up	N-desmethylalmotriptan	-
M223T123	2.048	0.009	2.426	up	5,8-Tetradecadienoic acid	-
M226T513	2.524	0.003	3.065	up	Nitroglycerin	-
M239T76	2.572	0.044	2.365	up	L-Menthyl acetoacetate	-
M249T137	2.284	0.003	2.554	up	Norambreinolide	-
M251T152	2.003	0.007	2.331	up	7Z,10Z-Hexadecadienoic acid	-
M267T243	2.231	0.033	2.273	up	Stearaldehyde	-
M277T163	2.353	0.006	2.557	up	Alpha-Linolenic acid	-
M277T29	2.193	0.022	2.311	up	Glycerol 1-propanoate diacetate	-
M277T31_1	2.262	0.002	2.808	up	Alcophosphamide	-
M282T29	0.476	0.015	2.610	down	Glutaminylhistidine	-
M286T127	0.414	0.001	3.059	down	N-[2-(4-Prenyloxyphenyl)ethyl]tiglamide	-
M286T28	4.756	0.000	4.382	up	Rutaecarpine	-
M292T561	3.141	0.000	3.394	up	Nonivamide	-
M297T570_2	3.567	0.017	2.873	up	3-Oxooctadecanoic acid	-

M298T192	4.204	0.012	3.263	up	Sphingosine	-
M305T32	5.343	0.000	4.195	up	Pelargonidin	-
M314T152	0.449	0.000	2.997	down	4-Oxoretinal	-
M315T173	2.422	0.026	2.184	up	Pregnenolone	-
M317T105	2.240	0.012	2.529	up	Leukotriene A4	-
M323T30_2	2.218	0.030	2.436	up	Mulberrofuran S	-
M324T198	3.017	0.021	2.772	up	N-Oleoylethanolamine	-
M326T217	12.217	0.002	4.644	up	Stearoylethanolamide	-
M341T29	3.079	0.001	3.410	up	2-O-alpha-D-Galactopyranosyl-1-deoxynojirimycin	-
M352T221	2.689	0.040	2.543	up	Docosadienoate (22:2n6)	-
M359T192	4.187	0.012	3.364	up	Tetracosatetraenoic acid (24:4n-6)	-
M360T198	3.396	0.033	2.841	up	16b-Hydroxystanozolol	-
M374T202	3.632	0.007	3.244	up	Adrenoyl ethanolamide	-
M378T29_2	2.025	0.006	2.338	up	Chondroitin	-
M378T566	0.417	0.002	2.854	down	2-(Arabinosylamino)-3-(glucosylamino)propanenitrile	-
M381T29	0.445	0.008	2.718	down	Eletriptan	-
M384T198	3.309	0.017	3.015	up	Pentadecanoylcarnitine	-
M385T198	3.191	0.011	2.798	up	1-Phenyl-1,3-eicosanedione	-
M386T217	12.964	0.002	4.777	up	Diethylhexyl adipate	-
M387T217	9.191	0.002	4.501	up	3-Hydroxy-1-phenyl-1-eicosanone	-
M395T29	0.384	0.003	3.001	down	Chondroitin	-
M401T243	2.144	0.045	2.198	up	Bis(2-methylundecan-2-yl) Disulfide	-
M410T206	2.274	0.028	2.345	up	DG(i-20:0/a-25:0/0:0)	-
M419T352	2.018	0.049	1.987	up	3-[(1E)-1-{4-[2-(dimethylamino)ethoxy]phenyl}-1-(3-hydroxyphenyl)but-1-en-2-yl]phenol	-
M429T28	6.110	0.008	4.032	up	3-phenyl-1-[2,4,6-trihydroxy-3,5-bis(3-methylbut-2-en-1-yl)phenyl]propan-1-one	-

M430T29	0.421	0.002	2.690	down	Ochratoxin C	-
M431T565	2.686	0.000	2.815	up	13'-Hydroxy-gamma-tocopherol	-
M434T202	5.177	0.009	3.449	up	11'-Carboxy-alpha-chromanol	-
M435T202	2.022	0.048	2.307	up	Demethylphylloquinone	-
M447T295	0.394	0.018	2.425	down	4-Hydroxy-5-(3',4',5'-trihydroxyphenyl)-valeric acid-O-methyl-O-glucuronide	-
M449T296	0.411	0.017	2.432	down	Flufenprox	-
M465T29	3.511	0.002	3.534	up	Corchorusoside E	-
M466T221	2.114	0.030	2.155	up	Arnamiol	-
M555T191	3.994	0.029	3.006	up	Cer(t18:0/16:0)	-
M581T196	8.038	0.008	4.002	up	FAHFA(18:1(9Z)/12-O-18:0)	-
M611T217	16.249	0.004	4.865	up	Ethylene glycol distearate	-
M671T296	0.446	0.044	2.034	down	4-[N-(p-Coumaroyl)serotonin-4''-yl]-N-feruloylserotonin	-
M681T296	0.446	0.028	2.181	down	Myricanol 5-laminaribioside	-
M682T296	0.470	0.025	2.149	down	6-{[2-(2,4-dihydroxyphenyl)-3-(3,7-dimethylocta-2,6-dien-1-yl)-7-hydroxy-8-(3-methylbut-2-en-1-yl)-4-oxo-4H-chromen-6-yl]oxy}-3,4,5-trihydroxyoxane-2-carboxylic acid	-
M697T295	0.399	0.025	2.332	down	3,4,5-trihydroxy-6-{[3-(2-methyl-1,3-thiazol-4-yl)-2-[(16R)-5,7,11-trihydroxy-8,8,10,12,16-pentamethyl-9-oxo-17-oxa-4-azabicyclo[14.1.0]heptadec-4-en-3-yl]prop-2-en-1-yl]oxy}oxane-2-carboxylic acid	-
M711T296	0.489	0.029	2.060	down	Nomilinic acid 17-glucoside	-
M729T284	0.388	0.013	2.761	down	PC(14:0/18:2(9Z,12Z))	-
M755T287	0.398	0.030	2.454	down	PC(14:0/20:3(5Z,8Z,11Z))	-
M757T303	0.450	0.002	2.706	down	PC(14:0/20:2(11Z,14Z))	-
M775T268	0.358	0.006	2.880	down	PC(14:1(9Z)/22:6(4Z,7Z,10Z,13Z,16Z,19Z))	-
M779T300	0.405	0.008	2.846	down	Ganglioside GM1 (d18:1/14:0)	-
M791T244	2.034	0.008	2.563	up	PG(16:1(9Z)/22:6(4Z,7Z,10Z,13Z,16Z,19Z))	-
M791T259	0.304	0.004	3.327	down	CL(18:0/18:0/18:0/22:5(4Z,7Z,10Z,13Z,16Z))	-

M797T414	0.433	0.002	2.582	down	PC(20:1(11Z)/P-18:1(11Z))	-
M799T258	0.444	0.004	2.887	down	3,4,5-trihydroxy-6-{[7-hydroxy-2-(3-hydroxy-4-methoxyphenyl)-4-oxo-3,8-bis[3,4,5-trihydroxy-6-(hydroxymethyl)oxan-2-yl]-4H-chromen-5-yl]oxy}oxane-2-carboxylic acid	-
M800T258	0.444	0.004	2.884	down	Delphinidin 3-lathyroside 5-(6-acetylglucoside)	-
M801T271	0.448	0.019	2.557	down	PC(18:3(6Z,9Z,12Z)/20:5(5Z,8Z,11Z,14Z,17Z))	-
M803T288	0.388	0.018	2.674	down	PC(16:1(9Z)/22:6(4Z,7Z,10Z,13Z,16Z,19Z))	-
M804T304	0.367	0.013	2.849	down	Dulcoside A	-
M805T288	0.409	0.010	2.723	down	Lactosylceramide (d18:1/12:0)	-
M805T304	0.364	0.015	2.982	down	Licoricesaponin C2	-
M817T262	0.423	0.005	2.776	down	Majoroside F2	-
M828T414	0.385	0.001	2.771	down	PC(16:1(9Z)/22:2(13Z,16Z))	-
M830T258	0.452	0.001	2.861	down	Alatanin 2	-
M832T414	0.459	0.003	2.567	down	PC(22:6(4Z,7Z,10Z,13Z,16Z,19Z)/P-18:1(11Z))	-
M833T414	0.460	0.000	2.675	down	PS(15:0/24:0)	-
M854T293	0.357	0.012	3.067	down	CDP-DG(a-13:0/i-12:0)	-
M854T309	0.407	0.030	2.895	down	Licoricesaponin G2	-
M858T569	2.375	0.003	2.543	up	6-{6-[6-(2,4-dihydroxyphenyl)-2-{4-[(E)-2-(2,4-dihydroxyphenyl)ethenyl]-2,6-dihydroxyphenyl}-4-(hydroxymethyl)cyclohex-3-ene-1-carbonyl]-3-hydroxy-2-(3-methylbut-2-en-1-yl)phenoxy}-3,4,5-trihydroxyoxane-2-carboxylic acid	-
M864T440	0.411	0.002	2.678	down	Ubiquinol-10	-
M896T440	0.399	0.000	2.994	down	TG(18:3(6Z,9Z,12Z)/20:5(5Z,8Z,11Z,14Z,17Z)/18:3(6Z,9Z,12Z))	-
M910T300	3.209	0.000	3.678	up	PS(DiMe(11,3)/DiMe(11,5))	-
M919T393	2.251	0.001	2.683	up	Phaseoloside D	-
M929T313	6.283	0.000	4.713	up	PI(18:0/22:5(4Z,7Z,10Z,13Z,16Z))	-
M935T304	2.460	0.001	3.224	up	PS(DiMe(11,5)/MonoMe(13,5))	-

M938T323	2.590	0.000	3.353	up	PS(DiMe(11,3)/DiMe(13,5))	-
M953T312	6.998	0.000	4.899	up	1,2-Di-(9Z,12Z,15Z-octadecatrienoyl)-3-(Galactosyl-alpha-1-6-Galactosyl-beta-1)-glycerol	-
M133T101	5.876	0.025	3.501	up	Eplerenone	+
M145T34	2.330	0.016	3.327	up	2-Bromoacetaldehyde	+
M152T30	0.469	0.009	2.809	down	(S)-Ureidoglycolic acid	+
M165T33	0.385	0.013	3.013	down	5-Ethyl-3-hydroxy-4-methyl-2(5H)-furanone	+
M175T36	2.089	0.000	3.063	up	L-Arginine	+
M185T30	2.728	0.000	3.575	up	N-(3-acetamidopropyl)pyrrolidin-2-one	+
M194T33	5.824	0.000	4.404	up	4-Phenylpyridine	+
M236T34	0.291	0.000	3.898	down	L-Aspartyl-4-phosphate	+
M246T30	2.055	0.009	2.523	up	Dezocine	+
M246T76	0.386	0.000	3.433	down	S-3-oxodecanoyl cysteamine	+
M259T33	2.266	0.015	2.514	up	2,4-Dichlorophenoxyacetic acid	+
M285T32	12.763	0.007	4.868	up	Dimethylallylpyrophosphate	+
M286T38	4.281	0.000	3.882	up	2-[(7-hydroxy-1-oxo-1H-isochromen-3-yl)formamido]acetic acid	+
M288T37_1	2.147	0.004	2.551	up	2-{[hydroxy(4-methoxy-1-benzofuran-5-yl)methylidene]amino}acetic acid	+
M289T29	2.543	0.026	2.910	up	Methionyl-Threonine	+
M290T35	0.484	0.001	2.802	down	Tiglylcarnitine	+
M299T237	2.659	0.027	2.601	up	Artemether	+
M299T38	0.148	0.016	3.989	down	LysoPA(8:0/0:0)	+
M300T192	4.840	0.013	3.524	up	Sphingosine	+
M300T38	0.132	0.009	4.171	down	Arabinopyranobiose	+
M301T29	2.736	0.000	3.561	up	(x)-2-Heptanol glucoside	+
M302T30	2.350	0.001	3.213	up	Sibutramine	+
M307T35	53.895	0.023	5.220	up	(1aalpha,2beta,3alpha,11calpha)-1a,2,3,11c-Tetrahydro-6,11-dimethylbenzo[6,7]phenanthro[3,4-b]oxirene-	+

					2,3-diol	
M316T153	0.475	0.000	3.071	down	4-Oxoretinal	+
M319T40	2.167	0.002	3.157	up	Leukotriene A4	+
M326T198	3.770	0.018	3.458	up	N-Oleoylethanolamine	+
M328T217	17.732	0.003	5.454	up	Stearoylethanolamide	+
M343T243	2.131	0.031	2.485	up	MG(P-18:0e/0:0/0:0)	+
M345T242	2.028	0.041	2.377	up	MG(18:0e/0:0/0:0)	+
M351T33	3.390	0.004	3.382	up	Penicillin V	+
M356T241	3.451	0.008	3.479	up	Erucic acid	+
M356T31	4.848	0.000	4.647	up	Robenidine	+
M360T80	2.224	0.001	3.014	up	2-Hydroxylauroylcarnitine	+
M367T224	2.471	0.011	2.893	up	Dihomo-gamma-Linolenoyl ethanolamide	+
M400T164	2.181	0.000	2.854	up	L-Palmitoylcarnitine	+
M400T268	2.072	0.042	2.183	up	Pentacosanoic acid	+
M401T164	2.077	0.000	2.776	up	7-Ketocholesterol	+
M402T33	2.269	0.015	2.906	up	S-(1,2-Dichlorovinyl)glutathione	+
M416T144	2.728	0.001	3.427	up	3-Hydroxyhexadecanoylcarnitine	+
M428T189	2.465	0.001	3.170	up	Stearoylcarnitine	+
M429T189	2.137	0.000	2.942	up	Cholesteryl acetate	+
M437T33_1	2.382	0.000	3.278	up	[2-hydroxy-5-(3,5,6,7-tetrahydroxy-4-oxo-4H-chromen-2-yl)phenyl]oxidanesulfonic acid	+
M442T152	2.374	0.001	3.096	up	3-Hydroxy-11Z-octadecenoylcarnitine	+
M477T230_1	0.475	0.007	2.429	down	Ubiquinone-4	+
M523T574	2.411	0.040	2.527	up	Tuftsin	+
M584T567	2.805	0.001	3.320	up	N-(2R-Hydroxyhexadecanoyl)-2S-amino-9-methyl-4E,8E-octadecadiene-1,3R-diol	+
M671T258	0.380	0.001	3.661	down	Bromocriptine	+

M672T258_2	0.471	0.001	3.127	down	Citbismine F	+
M672T488	2.503	0.007	2.875	up	CE(20:5(5Z,8Z,11Z,14Z,17Z)	+
M687T258	0.404	0.001	3.443	down	Secoisolariciresinol 9,9'-diglucoside	+
M687T559	2.377	0.017	2.823	up	Ceramide (d18:1/25:0)	+
M688T152	6.052	0.000	4.958	up	Jubanine C	+
M699T411	0.475	0.031	2.404	down	Erythrodiol 3-palmitate	+
M727T258	0.474	0.000	3.043	down	Paradisin C	+
M728T415	0.387	0.015	2.779	down	CE(22:2(13Z,16Z))	+
M773T241_2	2.066	0.013	2.616	up	Oleanolic acid 3-[glucosyl-(1->4)-xyloside]	+
M815T414	0.377	0.002	3.077	down	PE(24:0/P-18:1(11Z))	+
M816T414	0.404	0.003	2.921	down	Sitoindoside I	+
M817T414	0.449	0.002	2.794	down	PE(24:0/P-18:0)	+
M831T335	2.080	0.029	2.609	up	N-Monodemethyl roxithromycin	+
M842T429	0.459	0.020	2.473	down	PA(21:0/22:0)	+
M854T465	0.339	0.032	2.617	down	TG(15:0/22:5(4Z,7Z,10Z,13Z,16Z)/15:0)	+
M857T414	0.457	0.003	2.718	down	Isofucosterol 3-O-[6-O-(9-Octadecenoyl)-b-D-glucopyranoside]	+
M858T414	0.482	0.001	2.788	down	TG(14:0/20:1(11Z)/15:0)	+
M878T480	2.356	0.046	2.545	up	TG(18:3(9Z,12Z,15Z)/18:1(11Z)/18:3(9Z,12Z,15Z))	+
M883T440	0.371	0.002	3.031	down	PC(o-22:0/22:3(10Z,13Z,16Z))	+
M886T440	0.465	0.002	2.790	down	Coenzyme Q10	+
M888T440	0.393	0.002	2.976	down	Ubiquinol-10	+
M926T440	0.487	0.000	2.634	down	TG(18:1(11Z)/18:0/18:1(11Z))	+
M929T296	7.184	0.000	5.231	up	3-O-Sulfogalactosylceramide (d18:1/24:1(15Z))	+
M998T465	2.109	0.033	2.261	up	TG(22:6(4Z,7Z,10Z,13Z,16Z,19Z)/20:5(5Z,8Z,11Z,14Z,17Z)/22:6(4Z,7Z,10Z,13Z,16Z,19Z))	+
M999T273	2.754	0.000	3.598	up	Ziziphin	+

Table S4 Differential lipid species Cela/tMCAO in mouse brain, according to significance evaluation and VIP values

ID	Fold change	p.value	VIP	Regulated	Metabolite	Ion mode
M103T33	0.445	0.014	3.044	down	2-Hydroxybutyric acid	-
M173T29	2.086	0.036	2.503	up	1-[(2,5-Dimethylphenyl)azo]-2-naphthalenol	-
M255T26	0.439	0.034	3.181	down	Dodecamethylpentasiloxane	-
M269T189	0.441	0.018	2.828	down	3-Oxohexadecanoic acid	-
M345T52	2.926	0.000	4.979	up	Cortexolone	-
M403T43	2.139	0.000	3.847	up	1alpha-O-Methylquassin	-
M405T52	3.013	0.000	5.025	up	Annoglabasin C	-
M533T578	0.083	0.036	4.725	down	1-Linoleoylglycerophosphocholine	-
M727T258	2.015	0.016	2.145	up	C.I. Acid Violet 49	-
M861T497	0.439	0.033	2.962	down	PA(22:0/24:0)	-
M910T300	0.472	0.001	3.322	down	PS(DiMe(11,3)/DiMe(11,5))	-
M929T313	0.463	0.003	2.859	down	PI(18:0/22:5(4Z,7Z,10Z,13Z,16Z))	-
M953T312	0.378	0.001	3.537	down	1,2-Di-(9Z,12Z,15Z-octadecatrienoyl)-3-(Galactosyl-alpha-1-6-Galactosyl-beta-1)-glycerol	-
M194T33	0.471	0.036	3.733	down	4-Phenylpyridine	+
M236T34	2.469	0.027	3.156	up	L-Aspartyl-4-phosphate	+
M280T29_1	0.371	0.011	3.984	down	Glutamylaspartic acid	+
M307T35	0.017	0.019	7.207	down	(1aalpha,2beta,3alpha,11calpha)-1a,2,3,11c-Tetrahydro-6,11-dimethylbenzo[6,7]phenanthro[3,4-b]oxirene-2,3-diol	+
M356T241	0.317	0.014	3.976	down	Erucic acid	+
M449T570	0.488	0.029	2.713	down	4alpha-Formyl-4beta-methyl-5alpha-cholesta-8,24-dien-3beta-ol	+

M492T242	8.478	0.049	3.821	up	N-hexacosanoylglycine	+
M561T278_1	0.333	0.045	3.209	down	Cucurbitacin C	+
M655T368	0.490	0.012	3.159	down	DG(18:3(6Z,9Z,12Z)/20:5(5Z,8Z,11Z,14Z,17Z)/0:0)	+
M672T488	0.476	0.026	3.472	down	CE(20:5(5Z,8Z,11Z,14Z,17Z)	+
M687T559	0.458	0.023	3.784	down	Ceramide (d18:1/25:0)	+
M688T152	0.392	0.003	3.687	down	Jubanine C	+
M878T480	0.426	0.043	3.072	down	TG(18:3(9Z,12Z,15Z)/18:1(11Z)/18:3(9Z,12Z,15Z))	+
M923T294	0.492	0.001	3.720	down	PS(DiMe(11,3)/DiMe(13,5))	+
M929T296	0.410	0.006	3.350	down	3-O-Sulfogalactosylceramide (d18:1/24:1(15Z))	+
M941T305	0.463	0.029	3.261	down	3-O-Sulfogalactosylceramide (d18:1/26:1(17Z))	+

Table S5 The enriched KEGG pathway of differential metabolites of Cela/Sham in mouse plasma

Pathwayname	p-value	Metabolite	Impact
Sphingolipid metabolism	4.57E-05	Sphingomyelin;Digalactosylceramide;Glucosylceramide; Sulfatide	0.08
Arachidonic acid metabolism	0.005	5,6-EET; Phosphatidylcholine; 14,15-DHET	0.02
Linoleic acid metabolism	0.05	Phosphatidylcholine	0
Glycerophospholipid metabolism	0.05	Phosphatidylethanolamine; Phosphatidylcholine	0.2
Valine, leucine and isoleucine biosynthesis	0.082083	L-Isoleucine	0
alpha-Linolenic acid metabolism	0.13013	Phosphatidylcholine	0
Glycosylphosphatidylinositol (GPI)-anchor biosynthesis	0.13945	Phosphatidylethanolamine	0
Terpenoid backbone biosynthesis	0.17581	Dimethylallyl diphosphate	0
Biosynthesis of unsaturated fatty acids	0.32232	(4Z,7Z,10Z,13Z,16Z,19Z)-Docosahexaenoic acid	0
Valine, leucine and isoleucine degradation	0.35138	L-Isoleucine	0
Steroid biosynthesis	0.36546	3alpha,7alpha-Dihydroxy-5beta-cholestane	0
Primary bile acid biosynthesis	0.39278	3alpha,7alpha-Dihydroxy-5beta-cholestane	0.03

Aminoacyl-tRNA biosynthesis	0.40602	L-Isoleucine	0

Table S6 The enriched KEGG pathway of differential metabolites of Cela/Sham in mouse brain

**Pathwayname**	**p-value**	**Metabolite**	**Impact**
Arachidonic acid metabolism	0.0054649	5,6-EET;Phosphatidylcholine; Leukotriene A4;14,15-DHET	0.101
Glycerophospholipid metabolism	0.0054649	Phosphatidylethanolamine; Phosphatidylcholine;1-Acyl-sn-glycero-3-phosphocholine; Phosphatidylserine	0.26332
Sphingolipid metabolism	0.067564	Sphingomyelin; Sulfatide	0
Linoleic acid metabolism	0.098897	Phosphatidylcholine	0
Phosphonate and phosphinate metabolism	0.11751	2-Aminoethylphosphonate	0
Valine, leucine and isoleucine biosynthesis	0.15362	L-Isoleucine	0
alpha-Linolenic acid metabolism	0.23775	Phosphatidylcholine	0
Glycosylphosphatidylinositol (GPI)-anchor biosynthesis	0.25358	Phosphatidylethanolamine	0.00399
Terpenoid backbone biosynthesis	0.31377	Dimethylallyl diphosphate	0
Fatty acid degradation	0.56031	L-Palmitoylcarnitine	0
Valine, leucine and isoleucine degradation	0.5696	L-Isoleucine	0
Tryptophan metabolism	0.5787	5-Hydroxykynurenamine	0.0139
Steroid biosynthesis	0.58762	5-Hydroxykynurenamine	0
Primary bile acid biosynthesis	0.62149	3alpha,7alpha-Dihydroxy-5beta-cholestane	0.02724
Aminoacyl-tRNA biosynthesis	0.6374	L-Isoleucine	0
Steroid hormone biosynthesis	0.80676	Dehydroepiandrosterone sulfate	0

Table S7 Frequency of lipid metabolites and the involved KEGG pathway

Metabolites	Frequency	Pathway	Synonyms in HMDB
Phosphatidylcholine	18	Glycerophospholipid metabolism;alpha-Linolenic acid metabolism; Linoleic acid metabolism;Arachidonic acid metabolism	PC(14:0/18:2(9Z,12Z))
Phosphatidylethanolamine	9	Glycerophospholipid metabolism;Glycosylphosphatidylinositol (GPI)-anchor biosynthesis	PE(24:0/P-18:1(11Z))
Sulfatide	6	Sphingolipid metabolism	3-O-Sulfogalactosylceramide (d18:1/24:1(15Z))
L-Isoleucine	8	Valine, leucine and isoleucine degradation; Aminoacyl-tRNA biosynthesis	L-Isoleucine
Sphingosine	4	Sphingolipid metabolism	Sphingosine
(9Z,12Z,15Z)-Octadecatrienoic acid	4	alpha-Linolenic acid metabolism; Biosynthesis of unsaturated fatty acids	Alpha-Linolenic acid
Dimethylallyl diphosphate	4	Terpenoid backbone biosynthesis	Dimethylallylpyrophosphate
Cholesterol ester	3	Steroid biosynthesis	CE(20:5(5Z,8Z,11Z,14Z,17Z)
L-Arginine	3	Arginine biosynthesis;Arginine and proline metabolism;Aminoacyl-tRNA biosynthesis	L-Arginine
L-Palmitoylcarnitine	3	Fatty acid degradation	L-Palmitoylcarnitine
5-Hydroxykynurenamine	3	Tryptophan metabolism;Steroid biosynthesis	5-Hydroxykynurenamine
3alpha,7alpha-Dihydroxy-5beta-cholestane	3	Steroid biosynthesis;Primary bile acid biosynthesis	3a,7a-Dihydroxy-5b-cholestane
N-Acylsphingosine	2	Sphingolipid metabolism	Ceramide (d18:1/25:0)
2-Hydroxybutanoic acid	2	Propanoate metabolism	2-Hydroxybutyric acid
N-Acetyl-L-aspartate	2	Alanine, aspartate and glutamate metabolism	N-Acetyl-L-aspartic acid
1-Acyl-sn-glycero-3-phosphocholine	2	Glycerophospholipid metabolism	1-Linoleoylglycerophosphocholine
(1aalpha,2beta,3alpha,11calpha)-1a,2,3,11c-Tetrahydro-6,11-	2	Metabolism of xenobiotics by cytochrome P450;Glycerophospholipid metabolism	(1aalpha,2beta,3alpha,11calpha)-1a,2,3,11c-Tetrahydro-6,11-

dimethylbenzo[6,7]phenanthro[3,4-b]oxirene-2,3-diol			dimethylbenzo[6,7]phenanthro[3,4-b]oxirene-2,3-diol
11-Deoxycortisol	2	Steroid hormone biosynthesis	Cortexolone
Lactosylceramide	2	Sphingolipid metabolism	Lactosylceramide (d18:1/12:0)
Pregnenolone	2	Steroid hormone biosynthesis	Pregnenolone
Phosphatidylserine	2	Glycerophospholipid metabolism	PS(18:0/22:5(7Z,10Z,13Z,16Z,19Z))
Sphingomyelin	2	Sphingolipid metabolism	SM(d18:1/18:0)
5,6-EET	2	Arachidonic acid metabolism	5,6-Epoxy-8,11,14-eicosatrienoic acid
14,15-DHET	2	Arachidonic acid metabolism	14,15-DiHETrE
